# Knowledge, Attitudes, and Perceptions Associated With Antimicrobial Stewardship Among Veterinary Students: A Multi-Country Survey From Nigeria, South Africa, and Sudan

**DOI:** 10.3389/fpubh.2020.517964

**Published:** 2020-10-21

**Authors:** Folorunso O. Fasina, Lerica LeRoux-Pullen, Peter Smith, Legesse K. Debusho, Aminu Shittu, Saleh M. Jajere, Oluwawemimo Adebowale, Ismail Odetokun, Michael Agbaje, Modupe M. Fasina, Olubunmi G. Fasanmi, Deborah van Dyk, Mohammed S. Abubakar, Monday M. Onakpa, Masaad G. Ali, Hozaifa S. Yousuf, Waliedin E. Elmgboul, Mohammed M. Sirdar

**Affiliations:** ^1^Emergency Centre for Transboundary Animal Diseases, Food and Agriculture Organisation of the United Nations, Dar es Salaam, Tanzania; ^2^Department of Veterinary Tropical Disease, University of Pretoria, Pretoria, South Africa; ^3^Department of Paraclinical Sciences, University of Pretoria, Pretoria, South Africa; ^4^Institute for Risk Assessment Sciences (IRAS), Utrecht University, Utrecht, Netherlands; ^5^Department of Production Animal Studies, University of Pretoria, Pretoria, South Africa; ^6^Department of Statistics, College of Science, Engineering and Technology, University of South Africa, Johannesburg, South Africa; ^7^Department of Veterinary Public Health and Preventive Medicine, Faculty of Veterinary Medicine, Usmanu Danfodiyo University, Sokoto, Nigeria; ^8^Department of Pathology and Microbiology, Faculty of Veterinary Medicine, Universiti Putra Malaysia, Seri Kembangan, Malaysia; ^9^Department of Veterinary Public Health and Preventive Medicine, Faculty of Veterinary Medicine, University of Maiduguri, Maiduguri, Nigeria; ^10^Department of Veterinary Public Health and Reproduction, College of Veterinary Medicine, Federal University of Agriculture, Abeokuta, Nigeria; ^11^Department of Veterinary Public Health and Preventive Medicine, Faculty of Veterinary Medicine, University of Ilorin, Ilorin, Nigeria; ^12^Department of Veterinary Microbiology and Parasitology, College of Veterinary Medicine, Federal University of Agriculture, Abeokuta, Nigeria; ^13^Department of Nursing Science, University of Pretoria, Pretoria, South Africa; ^14^Department of Animal Health, Federal College of Animal Health and Production Technology, Ibadan, Nigeria; ^15^Department of Veterinary Pathology, Faculty of Veterinary Medicine, Usmanu Danfodiyo University, Sokoto, Nigeria; ^16^Department of Veterinary Pharmacology, Faculty of Veterinary Medicine, University of Abuja, Abuja, Nigeria; ^17^Faculty of Veterinary Medicine, University of Khartoum, Khartoum, Sudan; ^18^Faculty of Veterinary Medicine, University of Albutana, Albutana, Sudan; ^19^Onderstepoort Veterinary Research, Agricultural Research Council, Pretoria, South Africa

**Keywords:** antimicrobial stewardship, antimicrobial resistance, Africa, veterinary training, AMR, prescribers, antimicrobial use

## Abstract

In African countries, antimicrobial resistance (AMR) issue remains pertinent. Despite this, little efforts have been made to assess the future veterinary prescribers on their knowledge, attitudes and practices (KAP) related to antimicrobial usage. This multi-country survey attempts to explore the KAP of future veterinarians on stewardship of antimicrobial and identify knowledge gaps. Eight veterinary schools participated from Nigeria, Sudan and South Africa. Data regarding perceptions and knowledge were analyzed using Chi-square χ^2^ test, Spearman's (Rho) Rank order correlation and factor analysis using principal component factoring extraction method. Fifty-two percent of the study participants were final year veterinary students, respectively, and majority (77.2%) had no previous knowledge of biomedical sciences. Majority age were 22–27 years (24.7 ± 2.8) 79% and multiple career fields post-graduation were preferred. Overall, poor perceptions and knowledge of antimicrobial stewardship were observed with variations among countries and only 36.3% (*n* = 123) of the students were confident in their ability to choose the ideal antimicrobial agents for a specific patient/group of animals. The majority of the final year students were confident of their knowledge regarding AMR (68%), making of Gram staining (69.2%) and in choosing the most ideal route for administering a specific antimicrobial (74.7%). The final year students had significantly (*p* < 0.05) higher confidence level for knowledge compared with the pre-final year students. Tetracyclines, penicillins, and sulphonamides represent the three most abused veterinary antimicrobials with similar ranking across countries. South African (69.7 ± 20.5) and Sudanese (68.1 ± 15.4) had significantly (*p* < 0.0001) higher mean scores compared to the Nigerian students (44.3 ± 6.8) in the student's ability to correctly match some specific antimicrobials against their classes but Nigerian students performed better in ranking antimicrobials. This survey revealed poor to average knowledge of antimicrobial stewardship among veterinary students with significant knowledge gaps across the countries. It is recommended that the relevant regulatory and standardization authorities should make concerted efforts and interventions to regularly review curricula to ensure the delivery of targeted formative and normative training, and improved lectures on antimicrobial usage and stewardship in order to improve the awareness and behaviors of future prescribers. The identified knowledge gaps of veterinary medical students on antimicrobial stewardship must be bridge to safeguard the future.

## Introduction

Antimicrobial-resistant organisms are emerging and spreading rapidly in humans, animals and the environment. They have become public and animal health threats of major concern globally. While newer antimicrobials are becoming scarcer, these resistant organisms are defying the current generations of essential medications for human and animal with severe health impacts (1, 2). Recent reports from the United Nations World Health Organization (WHO) and other sister global health agencies issued warnings on the rise of multi-drug resistant (MDR) pathogens; with suggestions that drug-resistant infections could cause 10 million deaths each year by 2050, result in catastrophic economic damage similar to the 2008–2009 global financial crisis, and that by 2030, antimicrobial resistance (AMR) could have forced up to 24 million people into extreme poverty, if no coordinated and multisectoral “One Health” approach is enforced to checkmate the global rise in MDR bacterial infections (https://news.un.org/en/story/2019/04/1037471). Another frightening report of the AMR from researchers of the University of York, established that hundreds of rivers in the world are contaminated with antimicrobials, which would potentially escalate the development of resistance among the environmental microflora with implications for human and animal health (3, 4). According to this study, even though contamination was also found in rivers from developed countries, majority of the contamination burden pointed toward rivers in Africa (Nigeria—Lagos) and other Asian countries (Bangladesh, Pakistan). For instance, in Bangladesh, one river was found to carry antibiotic (Metronidazole) levels about 300 times higher than is considered “safe” for the environment.

Numerous reports have been produced with emphasis on the importance of prudent use of antimicrobials in public and animal health and their concomitant effect in humans and the environment (1, 5–11), and empirical links have been established between microbial resistance in humans, animals and the environment (12–20). Despite the immediate and potential consequences and challenges associated with AMR, many countries (especially in the developing economies) have not step up actions enough to meet the challenges of (1) control efforts on rates of usage of antimicrobial in intensive animal (food animal) production and food systems; (2) surveillance for resistance organisms and (3) implementation of the global and national action plans on AMR (21). The political will include the cost of policy implementation often serves as major limitations to the implementation of such programmes in view of many competing priorities. In addition, certain countries are burdened by wars, natural disasters, severe limitations of funds and in some cases, industrial interests—primarily the pharmaceuticals and food—which may act independently or in consonance to sabotage efforts for economic gains in this regards. Yet, the professionals have critical roles to play in the prevention of development of AMR globally.

While the teachers of veterinary and medical disciplines are supposed to focus on relevant training to meet the current needs and prevent future resistance-related problems, it is also expected that the next generation of veterinary and human doctors, and environmental health experts must be willing to prudently use antimicrobials only under necessary and appropriate conditions. These future doctors, especially, however will not be able to make informed decision on antibiotics/antimicrobial therapy should their trainings on these medicaments be deficient in content and information (22). A recent evaluation of the medical students in selected schools in the USA had identified gaps in the stewardship, knowledge and perception toward antimicrobial usage and advocated for a more focused training (1). Similar challenges and gaps have been identified for veterinary students in Australia (22) or in cross-disciplinary studies (23–25).

Specific calls have been made to professionals for the prudent and responsible use and prescription of antimicrobials (26), effective stewardship of use (26), and emphasis have been placed on regular evaluation of veterinary education and training at both undergraduate and postgraduate levels as well as focused research on public health aspects of antimicrobials (5, 9, 25, 26). In Africa, little efforts has been documented on the effects of veterinary training on antimicrobial usage among animal health professionals (27) despite the previous request to consider the global trend in packaging competent and relevant training for the doctors of the future (28). In this work, we conducted a multi-country survey of knowledge gaps, attitudes and perception of future African veterinarians in the use of antimicrobials with a view to identify and bridge training gaps identified. Specifically, it is hoped that the outcome could influence curriculum design on antimicrobial stewardship and policy implementation in African veterinary institutions.

## Materials and Methods

The deans of 64 veterinary schools were contacted electronically through official and personal email as provided on the OIE website. A presentation was also made to 11 deans at the Third Annual OIE Regional Deans meeting for SADC, held on Monday, 17 February 2014 to promote the work. However, only 8 veterinary schools responded to the survey including 3 from Nigeria, 4 from Sudan and 1 from South Africa. It should be understood that school programmes were not harmonized across Africa and some of the school were on industrial actions or holidays during the time of the presentation of the questionnaire. The questionnaire was sent out in Arabic, English, French and Portuguese as relevant to each school. Inclusion criteria were the following: (1) respondent must be registered in an OIE recognized Veterinary Faculty in Africa (2) respondent must be in the pre-final or final year of veterinary medical degree, (3) respondent must have undergone training in veterinary microbiology, pharmacology and medicine as part of the undergraduate curriculum.

### Clearances and Ethical Statement

The study was commissioned by the Vice Chancellor's office and cleared for implementation by the Dean, Faculty of Veterinary Science (FVS). Questionnaire tool was approved by the Department of Production Animal Studies and the Department of Paraclinical Sciences, University of Pretoria. Permission to conduct the questionnaire survey among the student was granted by the Office of the Registrar, University of Pretoria and the Dean's office, FVS, and each student also gave consent to participate or refuse participation willingly and reserved the right to withdraw at any point in the survey. The study ensured that the privacy of each participants was adequately protected. Since no direct human or animal sample was obtained and only an academic questionnaire was involved, and because no individual was handled, specific ethical approval was waived.

### Study, Questionnaire Design, and Student Recruitment

A semi-quantitative questionnaire was designed at the Faculty of Veterinary Science, University of Pretoria to explore the level of knowledge and perception on AMR amongst pre-final and final year students of Veterinary Science (1, 27). The questionnaire was prepared and jointly reviewed by specialist veterinarians (pharmacologist, poultry specialist, pig specialist, production animal specialist, and a microbiologist). The questionnaire was pre-tested among fifteen selected second year veterinary students and adjusted appropriately. All pre-final and final year students were approached as indicated above. The subject and aim of the questionnaire was explained in the preliminary page and opportunity to participate willingly or opt out of participation was provided. Each student was given a questionnaire and allowed to fill it independently to prevent bias. Responses from each participating institution were coded using institutional and country identifiers. All data were entered into the Microsoft Excel spreadsheet (Microsoft Corporation, Redmond, Washington, USA). Three independent evaluators filtered the data, and datasets with significantly incomplete details or inconsistent responses were removed. A total of 353 responses were retained for analysis. The ranking of scores (1st, 2nd, 3rd, etc.) for each antibiotic was determined as the antimicrobial with the highest positive (right) responses based on frequency for students' perceptions for a particular question and following in that order, and where two antimicrobial agents have same frequency, they were ranked equally, and assigned the same score.

### Data Analysis

Characteristics and other variables related to the students were analyzed using descriptive statistics and presented as mean, percentages and proportions with 95% confidence intervals. The responses of the students on questions pertaining perceptions and knowledge of antimicrobial usage were merged before analysis as follows: for perceptions, “strongly agreed” and “agreed” responses were merged as single variable and “uncertain/not sure/neutral,” “disagree” and “strongly disagreed” were merged as one variable; while for knowledge, the response “confident” on each knowledge question was regarded as a single variable and others “unsure knowledge,” “vague idea of concepts” and “no idea of the concepts” related to antimicrobial stewardship were merged as single variable. These merged variables were subsequently analyzed and compared between the pre-final and final year students for all countries using χ^2^ analysis and *p-values*. Analysis was considered significant when *p* ≤ 0.05. Antimicrobial agents were independently ranked according to the degree of abuse based on students' perceptions and ranks were compared between countries using Spearman (Rho) Rank order correlation analysis. All data on perception (26 questions) and knowledge (10 questions) were thereafter analyzed using factor analysis using principal component factoring extraction method. Varimax Rotation Method with Kaiser Normalization (VRM-KN) was used for convergence of perceptions and knowledge on antimicrobials (29, 30). Reliability of the scales used in measuring perceptions and knowledge was based on a Cronbach's Alpha values of > 0.5. All statistical analyses were performed using Stata v9.0 (Stata Corporation, Lakeway Drive, College Station, Texas, USA).

In view of the fact that the work was a commissioned research for the Peer Enhanced Scholarship of Teaching and Learning (SoTL), University of Pretoria, South Africa, one of the key outputs for the research was to produce a specific report for the University of Pretoria, South Africa. Hence a university-level analyses of data were first made for South Africa using part of the pooled data (27). However, these analyses does not compromise the evaluation of the bigger data pool (for Africa) which were then re-analyzed for Africa, for purposes of country-level comparisons. The display of outputs was in the large part (≥ 80%) different from the earlier data published by Smith et al. (27) mentioned above.

## Results

### Demographic Characteristics of the Study Participants

A total of 105 (29.7%) respondents studied in Nigeria, 177 (50.1%) in Sudan and 71 (20.1%) in South Africa (Table 1) giving a total of 353 responses from eight schools of veterinary medicine. A total of 204 (57.8%) of the respondents were males and 145 (41.1%) were females with 184 (52.1%) and 163 (46.2%) representing final and pre-final year students, respectively. Majority 279 (79.0%) of these students were aged 22–27 (24.7 ± 2.8) years. The post-graduation preferred field of choices are poultry (26.6%), small animal practice (21.8%), mixed practice (21.0%), pharmaceutical industry (19.3%), State services (17.0%), and cattle practice (15.9%) among others (Table 1). Only 22.8% of all respondents have had previous knowledge in the field that may influence their response to the questionnaire. Averagely, each of the respondents fluctuates between 2 and 4 fields within the veterinary medical career. Based on class, minor to significant differences exist between proportions for the pre-final and final year students (Table 2).

**Table 1:** Baseline demography of the pre- and final-year Veterinary students (*n* = 353) sampled from eight Veterinary Faculties from Nigeria, South Africa and Sudan, 2014.

**Variables**	**Frequency (%)**
**Country**	
Nigeria	105 (29.7)
South Africa	71 (20.1)
Sudan^a^	177 (50.1)
**Class of veterinary students**^a^	
Pre-final year	163 (46.2)
Final year	184 (52.1)
**Gender**^b^	
Male	204 (57.8)
Female	145 (41.1)
**Age**^c^	
20–27	279 (79.0)
28–35	30 (8.5)
36–43	4 (1.1)
**Likely career choice post- graduation**^#^	
Small animal practice	77 (21.8)
Equine practice	42 (11.9)
Mixed practice	74 (21.0)
Feedlot	44 (12.5)
Dairy	36 (10.2)
Wildlife	31 (8.8)
Gross pathology	32 (9.1)
Pharmaceutical industry	68 (19.3)
State service	60 (17.0)
Beef Cattle	56 (15.9)
Sheep and goats	45 (12.8)
Pig	17 (4.8)
Poultry	94 (26.6)
Laboratory medicine/Clinical pathology	36 (10.2)
Exotic pet medicine	36 (10.2)
Education	32 (9.1)
Undecided	27 (7.7)
Other choices	32 (9.1)
**Previous knowledge in the field**^a^^*^	
No	268 (77.2)
Yes	79 (22.8)
**Overall**	**353 (100)**

a *6 missing data*;

b *4 missing data*;

c *40 missing data; Mean age of the study participants = 24.7 ± 2.8 years*;

# *the mean for number of likely career choice = 2 ± 2 (5 persons made no choice)*.

* *previous knowledge in the field meant that student has done previous studies at post-secondary school levels in pharmacology, biological research, microbiology or pharmacy, which may bias the opinion of respondents or influence the responses to antimicrobial-related questions*.

**Table 2:** Distribution of pre- and final-year Veterinary students sampled from eight Veterinary faculties of Nigeria, South Africa and Sudan, according to the measured variables, 2014.

	**Class of the Veterinary students**	
**Variables**	**Pre-final *n* (%)**	**Final-year *n* (%)**
**Country**^a^		
Nigeria	59 (36.2)	46 (25.0)
South Africa	42 (25.8)	29 (15.8)
Sudan	62 (38.0)	109 (59.2)
**Gender**^b^		
Male	82 (50.3)	119 (64.7)
Female	80 (49.1)	64 (34.8)
**Age**^c^		
20–27	129 (90.8)	146 (87.4)
28–35	11 (7.7)	19 (11.4)
36–43	2 (1.4)	2 (1.2)
**Overall**	**163 (100)**	**184 (100)**

a *6 missing data*,

b *4 missing data*,

c *40 missing data; Mean age of the study participants = 24.7 ± 2.8 years*.

### Perceptions of Antimicrobial Stewardship Among the Study Participants

As shown in Table 3, the survey revealed poor perceptions of antimicrobial stewardship among the students. Whereas, a total of 56.1% of the surveyed students agreed to have received formal training on rational use of antimicrobials, overall, > 50% of the students agreed/strongly agreed to 6 of the 26 questions on perceptions on antimicrobial usage (Table 3). Only about half agreed that AMR is an increasing global threat to human and animal health (*n* = 174; 50.7%), that the misuse of antimicrobials by veterinary practitioners contributes significantly to AMR (*n* = 172; 50.1%), that the misuse of antimicrobials by farmers contributes significantly to AMR (*n* = 175; 51.5%), that as individual veterinary practitioners, they can significantly contribute to preventing an increase in AMR (*n* = 172; 50%), and that cultures and antibiotic sensitivity testing should be done as frequently as possible to guide antimicrobial use (*n* = 171; 50.7%). Discouragingly, only 45.2% agreed that banning the use of prophylactic antimicrobials in food-producing animals will have a positive effect on decreasing the rise in AMR, 41.7% agreed that banning the use of antimicrobials as growth promoters in food-producing animals will have a positive effect on decreasing the rise in AMR and only 36.3% were confident in their ability to choose the ideal antimicrobial agents for a specific patient/group of animals (Table 3).

**Table 3:** Perception on antimicrobials of students who agreed or strongly agreed to the questions across eight Faculty of Veterinary Medicines, Africa, 2014.

**Variables**	**Strongly agreed/agreed**							
	**African countries**			**Veterinary class**				
	**Nigeria *n* (%)**	**Sudan *n* (%)**	**South Africa *n* (%)**	**Pre-final *n* (%)**	**Final *n* (%)**	**Overall *n* (%)**	**χ^2^**	***p*-value^a^**
1. Antimicrobial resistance is an increasing global threat to human and animal health	101 (98.1)	3 (1.8)	71 (100)	97 (55.7)	77 (44.3)	174 (50.7)	11.55	0.001^b^
2. The misuse of antimicrobials by veterinary practitioners contributes significantly to antimicrobial resistance	92 (89.3)	22 (12.6)	60 (84.5)	91 (52.9)	81 (47.1)	172 (50.1)	5.60	0.06
3. The misuse of antimicrobials by farmers contributes significantly to antimicrobial resistance	94 (93.1)	12 (7.0)	70 (98.6)	98 (56.0)	77 (44.0)	175 (51.5)	13.99	0.001^b^
4. The inappropriate use of antimicrobials in food-producing animals significantly contributes to antimicrobial resistance in human pathogens	89 (87.3)	17 (9.9)	43 (60.6)	84 (56.8)	64 (43.2)	148 (43.5)	9.89	0.002^b^
5. The inappropriate prescription of antimicrobials by human medical doctors is the main contributor to antimicrobial resistance in human pathogens	76 (74.5)	19 (11.1)	67 (94.4)	84 (51.9)	78 (48.1)	162 (47.5)	3.01	0.083^b^
6. I have received formal lectures on the rational use of antimicrobials during my under-graduate training	100 (98.0)	23 (13.2)	70 (98.6)	103 (53.6)	89 (46.4)	192 (56.1)	8.28	0.004^b^
7. My under-graduate training has prepared me well for making informed decisions when choosing an ideal antimicrobial for an individual patient	92 (90.2)	8 (4.6)	54 (76.1)	83 (53.9)	71 (46.1)	154 (44.9)	5.43	0.02^b^
8. As an individual in practice, I can significantly contribute to preventing an increase in antimicrobial resistance	99 (97.1)	13 (7.4)	60 (84.5)	98 (57.0)	74 (43.0)	172 (50.0)	15.14	<0.0001^b^
9. The misuse of antimicrobials was evident in the facilities where I have trained	41 (41.0)	67 (39.4)	17 (23.9)	53 (42.4)	72 (57.6)	125 (37.1)	1.40	0.237
10. Governing bodies in Africa are doing enough to help prevent a rise in antimicrobial resistance	25 (24.5)	74 (42.0)	1 (1.4)	42 (42.9)	56 (57.1)	100 (28.5)	0.74	0.381
11. Educating lay people on the importance of antimicrobials as controlled scheduled compounds will have a positive effect on decreasing the rise in antimicrobial resistance	85 (84.2)	10 (5.8)	62 (87.3)	84 (53.8)	72 (46.2)	156 (45.9)	5.81	0.016^b^
12. The use of antimicrobials in the food-producing animal industry (farm animals) contributes more to antimicrobials resistance than their use in companion animals	87 (85.3)	34 (19.8)	36 (50.7)	76 (48.7)	80 (51.3)	156 (45.7)	0.51	0.477
13. Banning the use of prophylactic antimicrobials in food-producing animals will have a negative effect on animal welfare	53 (52.0)	53 (31.9)	32 (45.1)	64 (46.7)	73 (53.3)	137 (40.9)	0.002	0.963
14. Banning the use of prophylactic antimicrobials in food-producing animals will have a positive effect on decreasing the rise in antimicrobial resistance	68 (68.7)	45 (26.6)	40 (56.3)	77 (50.7)	75 (49.3)	152 (45.2)	1.72	0.189
15. Banning the use of antimicrobials as growth promoters in food-producing animals will have a positive effect on decreasing the rise in antimicrobial resistance	74 (74.0)	27 (16.0)	41 (57.7)	71 (50.7)	69 (49.3)	140 (41.7)	1.31	0.252
16. Improved use of vaccines, biosecurity measures, and hygiene will decrease the need for antimicrobials in the food-producing industry	87 (86.1)	7 (4.1)	68 (95.8)	87 (53.7)	75 (46.3)	162 (47.9)	6.58	0.01^b^
17. Adhering to meat and milk withdrawal periods will help decrease the rise in antimicrobial resistance in human pathogens	94 (94.0)	6 (3.5)	48 (67.6)	80 (54.1)	68 (45.9)	148 (43.8)	5.65	0.017^b^
18. Broad-spectrum antimicrobials are ideal to use as first-line antimicrobials	62 (61.4)	61 (36.3)	27 (38.0)	83 (56.1)	65 (43.9)	148 (44.0)	9.91	0.002^b^
19. Third and fourth generation antimicrobials should only be used as a last resort in treatment	50 (51.0)	23 (14.0)	62 (87.3)	76 (56.3)	59 (43.7)	135 (40.9)	8.14	0.017^b^
20. Long-acting antimicrobials are more ideal for use in food-producing animals than shorter-acting equivalents	31 (30.7)	81 (47.9)	15 (21.1)	58 (46.4)	67 (53.6)	125 (37.1)	0.019	0.891
21. Cultures and antibiotic sensitivity testing (antibiograms should be done as frequently as possible to guide antimicrobial use)	94 (93.1)	8 (4.7)	69 (97.2)	91 (53.2)	80 (46.8)	171 (50.7)	5.07	0.024^b^
22. Financial constraints of animal owners in Africa disallow the use of cultures and antibiotic sensitivity testing e.g., antibiograms during an infection	77 (77.0)	21 (12.3)	55 (77.5)	82 (53.6)	71 (46.4)	153 (45.3)	5.74	0.017^b^
23. Drug legislation in Africa is on par with legislation in the rest of the world	40 (39.2)	82 (48.5)	13 (18.3)	66 (49.6)	67 (50.4)	133 (39.3)	0.587	0.444
24. I am confident that new classes of antimicrobials will be available in the near future to solve current resistance problems	57 (55.9)	20 (11.8)	5 (7.0)	37 (45.1)	45 (54.9)	82 (24.2)	0.187	0.665
25. The choice of an antimicrobial(s) by a veterinarian should largely be determined based on the cost implications to the farmers	71 (69.6)	51 (30.0)	14 (19.7)	63 (46.3)	73 (53.7)	136 (40.1)	0.070	0.792
26. I am confident in my ability to choose the ideal antimicrobial agents for a specific patient/group of animals in order to ensure optimal efficacy and safety	87 (86.1)	8 (4.7)	28 (39.4)	59 (48.0)	64 (52.0)	123 (36.3)	0.088	0.767

a *χ^2^ test*;

b *p < 0.05 refers to the significant statistical difference in the percentage/proportions between pre-final year and final-year veterinary students who strongly agreed/agreed to the questions regarding antimicrobial resistance*.

According to the countries surveyed, veterinary medical students from Sudan generally showed poorer perceptions on antimicrobial stewardship compared to those from veterinary schools in Nigeria and South Africa (Table 3). According to the year of studies in the surveyed African veterinary schools, veterinary students in the pre-final year got better scores in 17 of the 26 perception questions than the final year veterinary students. The perceptions of antimicrobials varied significantly between the pre-final and final year veterinary students across the countries (Table 3). For 13 of the 26 questions on perceptions, the pre-final year, compared to the final year students had significantly (*p* < *0.05*) higher proportions of those who strongly agreed/agreed to the questions representing relative poorer perceptions among the final year as shown in Table 3.

### Perceived Knowledge Toward Antimicrobial Stewardship Among the Study Participants

Overall, majority of the veterinary students were confident of their knowledge regarding antimicrobials as represented in Table 4. Unlike the questions on antimicrobial perceptions, majority of the veterinary medical students were confident on their knowledge regarding AMR (*n* = 223; 68%), making of Gram staining (*n* = 225; 69.2%) and of choosing the most ideal route for administering a specific antimicrobial (*n* = 248; 74.7%). Whereas, substantial proportion of the overall students had confident knowledge regarding choosing an alternative when the first choice of antimicrobial therapy failed (*n* = 198; 60%), choosing the desired time-frame for (duration of) therapy (*n* = 191; 58.2%) as well as finding reliable sources of information to guide empirical use of antimicrobials (*n* = 186; 57.1%). Only 48.1% of the overall students had confident knowledge related to the difference between time-dependent and concentration-dependent antimicrobials (Table 4).

**Table 4:** Perceived knowledge of antimicrobials of all participating pre-final and final-year Veterinary students, Faculty of Veterinary Medicines, Africa.

**Variables**	**Confident**							
	**African countries**			**Veterinary class**				
	**Nigeria** ***n* (%)**	**Sudan** ***n* (%)**	**South Africa** ***n* (%)**	**Pre-final** ***n* (%)**	**Final** ***n* (%)**	**Overall** ***n* (%)**	**χ^2^**	***p-value*^a^**
1. Spectrum, effect, distribution, indications, side effects, and contra-indications of the most commonly used antimicrobial classes in veterinary medicine, as well as the implication thereof	60 (61.2)	113 (68.1)	12 (16.9)	76 (41.5)	107 (58.5)	183 (55.3)	4.61	0.032^b^
2. The difference between time-dependent and concentration-dependent antimicrobials	30 (30.9)	84 (50.3)	47 (66.2)	71 (44.7)	88 (55.3)	159 (48.1)	0.58	0.446
3. Resistance mechanisms	68 (71.6)	135 (80.8)	23 (32.9)	104 (46.6)	119 (53.4)	223 (68.0)	0.028	0.868
4. Making a Gram-stain	85 (87.6)	101 (62.7)	40 (56.3)	95 (42.2)	130 (57.8)	225 (69.2)	6.92	0.009^b^
5. Interpreting antibiograms	54 (56.3)	68 (41.7)	45 (63.4)	67 (40.9)	97 (59.1)	164 (50.3)	4.90	0.027^b^
6. Finding reliable sources of information to guide empirical use of antimicrobials	50 (51.5)	104 (64.6)	35 (49.3)	84 (45.2)	102 (54.8)	186 (57.1)	0.75	0.386
7. Choosing the most ideal route for administering a specific antimicrobial	73 (76.0)	135 (79.9)	42 (59.2)	100 (40.3)	148 (59.7)	248 (74.7)	15.95	<0.0001^b^
8. Choosing the desired time-frame for (duration of) therapy	60 (62.5)	106 (64.2)	26 (36.6)	77 (40.3)	114 (59.7)	191 (58.2)	8.09	0.004^b^
9. Choosing an alternative if my first choice of antimicrobial therapy failed	55 (57.3)	123 (73.7)	21 (29.6)	82 (41.4)	116 (58.6)	198 (60.0)	6.13	0.013^b^
10. Designing an integrated treatment protocol for a specific animal with an infection which includes supportive therapy	55 (57.3)	113 (68.1)	18 (25.4)	70 (38.0)	114 (62.0)	184 (55.9)	13.78	<0.0001^b^

a ***χ**^2^ test*,

b *p < 0.05 refers to the significant statistical difference in the percentage/proportions of confidence regarding antimicrobial knowledge between pre-final year and final-year veterinary students*.

According to the countries surveyed, veterinary students from the three countries showed variable levels of confidence in knowledge of antimicrobial usage (Table 4). The level of knowledge regarding antimicrobial usage between the pre-final and final year students also varied significantly for 7 of the 10 questions, with the final year students having significantly (*p* < *0.05*) higher confidence level compared to the pre-final year students from all the countries (Table 4).

### Ranking on the Degree of Abuse of Antimicrobials According to the Student's Perceptions

**Figure 1:**
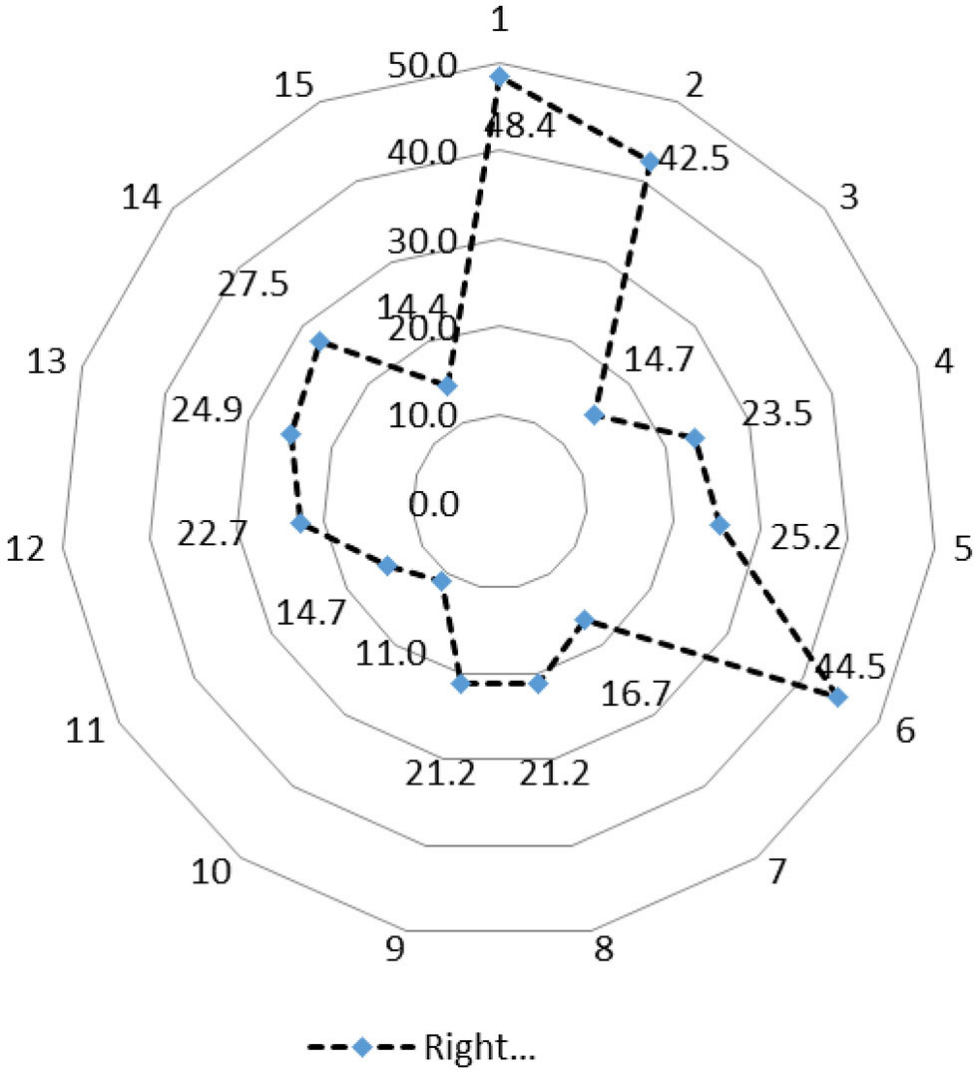
Spider-web analysis of knowledge of characteristics of individual antimicrobial agents by veterinary students from selected African schools (*n* = 353).

Based on students' perceptions across Africa, tetracyclines remain the most abused veterinary antimicrobials followed by penicillins, sulphonamides, macrolides, aminoglycosides, quinolones, amphenicols, polypeptides, cephalosporins, combination of antibiotics, and other medicaments in that order. Nigerian students' ranking was closest to that of Africa, Spearman (Rho) rank-order correlation coefficient rs = 0.98; *p* < 0.001, followed by South Africa, rs = 0.95; *p* < 0.001 and Sudan, rs = 0.78; *p* < 0.005 (Table 5). The spider-web analysis of knowledge of characteristics of individual antimicrobial revealed that the students did not score above 50% in any question (Figure 1; Box 1). Total correct mean matching score of 44.3, 68.1, and 69.7% were obtained for all the surveyed students from Nigeria, Sudan and South Africa, respectively (n = 353). South African and Sudanese students were able to correctly match more antimicrobials in their class than the Nigerian students (Table 6).

**Table 5:** Ranking on the degree of abuse of antimicrobials based on students' perceptions from eight Faculties of Veterinary Medicine, Africa.

**Antimicrobials**	**Ranking of abuse of antimicrobials based on students' perceptions**			
	**All** **(*n =* 352)**	**Nigeria** **(*n =* 105)**	**Sudan** **(*n =* 177)**	**South Africa** **(*n =* 70)**
Tetracyclines	1st	1st	1st	1st
Penicillins	2nd	2nd	2nd	2nd
Sulphonamides	3rd	3rd	3rd	3rd
Macrolides	4th	5th	8th	6th
Aminoglycosides	5th	4th	4th	4th
Quinolones	6th	6th	4th	7th
Amphenicols	7th	7th	10th	5th
Polypeptides	8th	9th	7th	8th
Cephalosporins	9th	8th	8th	9th
Combination of antimicrobials	10th	10th	6th	10th
Others	11th	11th	11th	11th

*Spearman (Rho) rank-order correlation coefficient (r_s_) = 0.98; p < 0.001 (Nigeria vs. Africa); Spearman (Rho) rank-order correlation coefficient (r_s_) = 0.78; p < 0.005 (Sudan vs. Africa); Spearman (Rho) rank-order correlation coefficient (r_s_) = 0.95; p < 0.001 (S. Africa vs. Africa); Spearman (Rho) rank-order correlation coefficient (r_s_) = 0.80; p < 0.005 (Sudan vs. Nigeria); Spearman (Rho) rank-order correlation coefficient (r_s_) = 0.96; p < 0.001 (South Africa vs. Nigeria). The strength and direction of ranking placed Nigeria as ranked closest to the generalized African ranking followed by South Africa and Sudan*.

Box 1 **Question on the knowledge of characteristics of individual antimicrobial agents by veterinary students**

Q1. Which one of the following antimicrobials should not be used in food-producing animals? **Answer: chloramphenicol**

Q2. Which one of the following bacteriostatic agents reaches bactericidal levels in the lungs? **Answer: Macrolides**

Q3. Which one of the following antimicrobial agents are not ideal to use for anaerobic bacteria? **Answer: Enrofloxacin**

Q4. Which one of the following antimicrobials would be most ideal to use in an immune-compromised patient? **Answer: Gentamycin**

Q5. The efficacy of aminoglycosides is mostly dependent on: Answer: **the magnitude of the total dose**

Q6. Penicillin is not effective against: Answer: ***Mycoplasma* spp**.

Q7. Penicillin G would likely be less effective in the following scenarios, EXCEPT: **Answer: early infections**

Q8. Which one of the following combinations is synergistic? **Answer: Ampicillin and amikacin**.

Q9. A loading dose is generally recommended for the following antibacterial agents: **Answer: Sulphonamides**

Q10. Which one of the following antibacterial drugs is not very effective against Gram-negative bacteria? **Answer: Erythromycin**

Q11. Which one of the following antibacterial drugs would be most ideal to use in the presence of pus and exudates? **Answer: Fluoroquinolones**

Q12. The long-acting characteristic of 2-pyrrolidone oxytetracycline is dependent on: **Answer: The route of administration**

Q13. Which one of the following drugs does not achieve prolonged antibacterial activity (> than 24 h)? **Answer: Doxycycline**

Q14. Which one of the following antibacterial agents is not time-dependent? **Answer: Nitro-imidazoles**

Q15. Which one of the following statements is not true for beta-lactamase inhibitors? **Answer: Resistance against amoxicillin-clavulanate is regarded as being extremely rare**

**Table 6:** Correct Matching of specific antimicrobials against their class (*n* = 353).

**Variable**	**Nigeria** **(*n =* 105) Percentage**	**Sudan** **(*n =* 164) Percentage**	**South Africa** **(*n =* 71) Percentage**	**χ^2^**	***P*-value**
1. Beta-lactams	43.8	68.0	81.4	9.9	<0.01
2. Penicillins	39.1	67.2	39.4	2.9	0.24
3. Cephalosporins	43.8	55.5	62.0	2.1	0.35
4. Tetracyclines	44.8	75.2	84.5	15.2	<0.001
5. Aminoglycosides	53.3	85.2	94.4	50.4	<0.0001
6. Macrolides	34.3	42.0	38.0	1.7	0.42
7. Amphenicols	41.9	70.0	62.0	3.2	0.20
8. Fluoroquinolones	42.9	65.5	77.5	8.8	0.01
9. Sulphonamides	41.0	56.1	63.4	2.1	0.35
10. Peptide antibiotics	58.1	96.1	95.8	128.9	<0.0001
**Total mean knowledge score**	**44.3** **±** **6.8**	**68.1** **±** **15.4**	**69.7** **±** **20.5**	**98.5**	** <0.0001**

Using the extracted factors analyzed with the principal component factoring extraction method and converged with VRM-KN, perceptions and knowledge on antimicrobials (*n* = 26; Table 3), students' perceptions on antimicrobials converged into five latent factors (categories) grouped as follows:

1. *Perception influenced by training received, clinical experience and sundry matters* (Questions 1, 21, 11, 3, 8, 6, 7, 17, 16, 2, 4, 26, 5, 22, 15, 12, 19, 14, *n* = 18);
2. *Perception based on legislation and costs* (Questions 10, 23, 24, 25, *n* = 4);
3. *Perception based on animal welfarism* (Question 13, *n* = 1);
4. *Perception based on idealism* (Questions 18, 20, *n* = 2); and
5. *Perception based on direct field observation* (Question 9, *n* = 1), Cronbach's Alpha values = 0.94, an indication of high reliability (matrix of factor loadings for the five factors is displayed in Supplementary Material).

Similarly, for the 10 questions listed to test students' depth of knowledge on antimicrobials (Table 4), there was convergence into four latent factors including: (1). *Knowledge that directly impact on therapeutic route, duration of administration and treatment protocol* (Questions 7, 8, 10, *n* = 3); (2). *Knowledge based on instructions and labels* (Questions 1, 2, 6, *n* = 3); (3). *Knowledge based on previous awareness of AMR* (Questions 3, 9, *n* = 2); and (4). *Knowledge linked with diagnostic technique* (Question 4, 5, *n* = 2), Cronbach's Alpha values = 0.67, an indication of moderate reliability (see Supplementary Material for matrix of factor loadings).

## Discussion

We have provided evidence on knowledge, attitudes and perceptions (KAP) and their effects on stewardship of antimicrobials in selected African countries. In the survey, we observed a higher proportion of male than the female students but note that country-level differences exist. In addition, the greater proportions of students in the field of veterinary medicine in Africa vary between 22 and 27 years, and will work post-graduation in areas of poultry, small animal practice, mixed practice, pharmaceutical industry, State services and cattle practice, indications that they will most-likely influence antimicrobial prescriptions and usage in food animal and pets. Little or scanty information exist on KAP of veterinary and medical professionals from African countries. It is also a fact that healthcare systems is “less” rigorously regulated in the region (31) and moreso, the global threats and burdens of emergence of antimicrobial resistant bacteria is increasing, particularly in the low-and-middle income countries (LMICs), particularly those from African and Asian countries (32–34). This study represents one of the rare continental attempts in the African region to assess the KAP of veterinary medical students, in this case, from Nigeria, South Africa and Sudan. It is expected that the findings from the present study will provide baseline data related to understanding the scope of AMR problems in the African context as well as identify knowledge gaps among veterinary medical professionals. The final year students had higher levels of knowledge regarding antimicrobial usage than the pre-final students, but generally there is poor KAP among both the final and final students surveyed across all the African countries.

Similar findings have been obtained in other reports among veterinary clinicians/students, medical and other non-medical students in Nigeria (35), Europe (24), USA (36), Australia (22), UAE (37), India (38), and Nepal (39).

### Perceptions Regarding Antimicrobial Stewardship

Overall, and irrespective of academic class or country, general perceptions regarding antibiotics among the veterinary medical students will appear poor (Table 3). Among the students, only 56.1% reported to have received formal lectures on the rational use of antimicrobials during the undergraduate training and 45.9% confirmed that educating lay people on AMR and controlled compounds could have positive effects on decreasing the rise in AMR; these emphasized some significant gaps in the veterinary medical schools' curricula in Africa. Based on the overall perceptions, only (1) the issues of AMR as an increasing global threat to human, animal and environmental health, (2) the misuse of antibiotics by veterinary practitioners and farmers will contribute to AMR, (3) the inappropriate use of antimicrobials in food-producing animals will significantly contribute to AMR in human pathogens, and (4) veterinary practitioners can significantly contribute to preventing an increase in AMR got good scores. These suggest that the views on the majority of issues that may increase the burden of antimicrobials are poorly perceived by the students who are future prescribers of these medicines. For example, 45.2% reported that banning the use of prophylactic antimicrobials in food-producing animals will have positive effect on decreasing AMR, and 41.7% agreed that banning antimicrobials as growth promoters in food-producing animals will have a positive effect on decreasing AMR which suggested that majority of these future prescribers may continue to use antimicrobials as growth promoters and for prophylactic/metaphylactic purposes. Approximately 50.7% will also subscribe to cultures and sensitivity to be done frequently for guiding antimicrobial usage, meaning that approximately half will prescribe and give antimicrobials without conducting susceptibility testing (Table 3). Combined, these responses revealed poor to low perceptions which may significantly impact on antibiotic usage among the pre-final and final veterinary medical students across all the countries surveyed.

Furthermore, only about 36.3% of the respondents believed they are confident of their ability to choose the ideal antimicrobials for a specific patient/group of animals in order to ensure optimal efficacy and safety. It was not surprising that in the 13 of the 26 questions related to perceptions, the pre-final had significantly (*p* < 0.05) higher “right” responses compared with the final year students. It can be inferred that for most of the questions on perceptions, that there is significant knowledge and perception gaps in and between the different classes of the students, a reflection of the embedded challenges in communicating the messages on AMR using the current veterinary curricular and courses. A previous study from 5 different veterinary schools in Nigeria (35) had earlier corroborated this finding. In that study, major knowledge gaps regarding AMR and antimicrobial stewardships among final-year veterinary students were identified and suggested an urgent need to improving antimicrobial perceptions in Nigerian veterinary schools. Another study (36) which assessed the antimicrobial use practices of veterinary clinicians at the University of Tennessee veterinary medical centre, found that clinicians who obtained their degree from 1970–1999 were more concerned about AMR compared to those from 2000–2009 and 2010–2016. This reflects less awareness on practices related to the judicious use of antimicrobials or undue familiarity and irresponsiveness to AMR issues by the later years' clinicians. Improving the practice of judicious antimicrobial use among veterinary students will be dependent on customized training that factor in stewardship, practice-oriented training in school through adaptation of proof of concept method of imparting knowledge and the use of problem-based learning in the training institutions (40).

### Knowledge Related to Antimicrobial Usage

Unlike the aspect of the study on perceptions, majority of the respondents were confident of their knowledge related to antibiotic usage with the final year demonstrating significantly higher knowledge levels compared to the pre-final year students (Table 4). Specifically, in 7 of the 10 knowledge questions, the final year students had significantly (*p* < *0.05*) high confidence levels compared to the pre-final year. About 68, 69.2, and 74.7% were confident on their knowledge regarding AMR, making Gram staining, and choosing the most ideal route for administering a specific antimicrobial, respectively (Table 4). Only 48.1% had reported confident knowledge related to the difference between time-dependent and concentration-dependent antimicrobials. Although, according to the results, the final year showed high knowledge levels, there are still some spaces to increase and improve on the knowledge of the veterinary medical students. In agreement to our findings, Jairoun et al. (37) found a significant knowledge gap in the medical curricula of the UAE medical institutes. In contrary, Huang et al. (41) found high knowledge levels related to antibiotic awareness and attitudes among Chinese medical students reflecting effectiveness of the Chinese medical curriculum on raising awareness. Abbo et al. (1) have similarly emphasized the need for intense education on the principles and practices of appropriate use of antibiotics and antimicrobial stewardships. Our findings call for urgent intervention in the veterinary medical curricula in the African veterinary medical schools. Interventions such as joint (African-wide) curricular review for harmonization and standardization, organization of well-packaged industry lectures for veterinary students, seminars and workshops including media campaign awareness could positively frame the minds of future veterinarians on their behaviors, attitudes and prescription practices regarding antimicrobials. Similar review and possible applications/interventions should be focused on the current medical curricula in medical schools.

### Degree of Abuse of Antimicrobials

The dearth of knowledge in the classification and characterization of antibiotics, and the perception especially in the degree of abuse of antimicrobials among veterinarians in training across Africa as shown by this study could contribute largely to the prevalence of AMR in livestock production in Africa, residue release in the human food chains, contaminations of the environment and their devastating effects. This can further endanger the therapeutic effectiveness of antibiotics, failure of treatments in livestock and subsequently in humans, and which could eventually lead to exorbitant cost of antibiotic therapy and high case fatality rate (42). The lack of knowledge on antimicrobial dosing; be it overdosing or under-dosing of antibiotics in the livestock industry is integral to and will consequently degenerate into the increase and spread of AMR (43). Through the perception of the veterinary students in Africa, tetracycline, penicillins, sulphonamides, macrolides, and aminoglycosides are the five most abused of all the antimicrobials used in Africa. This finding is in agreement with earlier studies in Ghana (44), South Africa (45), and Nigeria (46), and it should be a source of concern to stakeholders and the authorities. Regulatory authorities should intensify efforts to combat the continued misuse of these identified drugs and it becomes necessary to assess the situation in humans across Africa to see if the patterns are similar.

Furthermore, the study has highlighted that the veterinarians in training across Africa do not have sufficient knowledge on the different classes of antibiotics and their indications, a finding corroborated by similar findings from China (41) and the United Arab Emirates (37). To this end, this knowledge gap should be targeted for training and in compliance with the submission of the World Health Assembly (WHA), 2015, which endorsed a global action plan on AMR. Specifically, the WHA emphasized that public awareness should be increased and there should be an improvement in the understanding of AMR, which are key strategic objectives of a comprehensive plan on antimicrobial resistance. This report from World Health Organization thereby suggests the monitoring and educational interventions targeted at rationalizing the antibiotics prescription, disposal and consumption in order to control AMR (47). Considering all the aforementioned, there is also the need to aggressively tackle or approach the issue of AMR from the one health perspective (48) for effective and positive results.

We confirmed that perceptions were largely influenced primarily by the training and clinical experienced received during training while current knowledge among veterinary students is a factor of practice and instructions/labels. These understanding should form the basis for future programmes designed for training future veterinarians and medical doctors across Africa. It should be known that this work was subject to the limitation of numbers of schools that responded and participated in the survey (*n* = 8/64). This was possibly because an electronic system was used to dispatch the forms (with some limited paper trail), and several of the institutions does not regularly access internet. In addition, most school have unstable academic sessions during the survey period. While it is agreed that the small sample size (*n* = 353) may not be representative enough for all African countries, and there may be disparities in facilities available for learning, teaching methods and learning outcomes, because similar levels of competencies are expected from all veterinarians post-graduation, similar platform of evaluation should be utilized as we did in this study. We circulated the questionnaire to all institutions based on the details available on the OIE website for heads of all veterinary institutions in Africa. We also attended the Third Annual OIE Regional Deans Meeting to notify them of the survey and encourage participation. Furthermore, the survey was to target a subset of only the pre-final and final year students, hence it represented our best effort in conducting the survey. It was equally difficult to determine the exact sample size because Africa remain a data-scarce society and we did not know the exact number of veterinary students who should form the sample frame. Future studies should make effort to address these inherent challenges and make adequate budgetary provision to extend this study.

We are aware that significant socio-economic differences exist between the three countries included in this study. For instance, the GDP per capita (2018 current in US$) for the countries: Nigeria, South Africa and Sudan was US$ 2,028.20; US$ 6,374.00 and US$ 977.30, respectively (https://data.worldbank.org/indicator/NY.GDP.PCAP.CD?locations=ZG). This may have influenced to some degree the outcome of this study but whether this affected the outcome of this research was not investigated in this study.

It is our conclusion that almost all the spheres and fields tested in this work influenced the knowledge of and perception on antimicrobials, which are key determinants in the use and prescription of antimicrobial by future animal health officers. No knowledge field on antimicrobials should be left out in the training of veterinary students. Finally, differences exist in educational systems and resources used, and students do not believe that they have self-prepare enough to face the challenges of future prescriptions and stewardships of antimicrobials (1). The identified education gaps (formative and normative) should be addressed using some forms of standardization in curricula across Africa.

## Data Availability Statement

The raw data supporting the conclusions of this article will be made available by the authors, without undue reservation, to any qualified researcher.

## Author Contributions

FF, LL-P, PS, and MS: conceptualization. FF, LL-P, PS, LD, and MS: methodology and resources. AS, OA, IO, MA, MF, OF, DD, MSA, OM, MGA, HY, and WE: field investigation and data collection. FF, LL-P, LD, and MS: formal analysis. FF: writing—original draft preparation. FF, SJ, OA, IO, MA, MF, and OF: writing—review and editing. FF, LL-P, PS, and LD: supervision. FF, LL-P, PS, and MS: project administration. FF: funding acquisition. All authors: visualization.

## Conflict of Interest

The authors declare that the research was conducted in the absence of any commercial or financial relationships that could be construed as a potential conflict of interest.
