# Supplementary material for: Knowledge, Attitudes, and Perceptions Associated With Antimicrobial Stewardship Among Veterinary Students: A Multi-Country Survey From Nigeria, South Africa, and Sudan
Source: Front Public Health. 2020 Oct 21;8:517964. doi: 10.3389/fpubh.2020.517964 (PMC7609782; doi:10.3389/fpubh.2020.517964)
Supplement: Supplementary file 1 [file Table_1.DOCX]

**Appendix 1a. Factor extraction using principal component analysis (PCA) method and converged using Varimax Rotation Method with Kaiser Normalization (VRM-KN) for the 26-item questions on perceptions and knowledge on antimicrobials**

| **Reliability Statistics** | | | | | | | | | |  |  |
| --- | --- | --- | --- | --- | --- | --- | --- | --- | --- | --- | --- |
| Cronbach's Alpha | | | Cronbach's Alpha Based on Standardized Items | | | | N of Items | | |  |  |
| .940 | | | .930 | | | | 26 | | |  |  |
| **Item-Total Statistics** | | | | | | | | | | | |
|  | Scale Mean if Item Deleted | | | | Scale Variance if Item Deleted | | Corrected Item-Total Correlation | | Squared Multiple Correlation | | Cronbach's Alpha if Item Deleted |
| 4Q1 | 75.336 | | | | 494.727 | | .888 | | .892 | | .933 |
| 4Q2 | 75.461 | | | | 507.521 | | .781 | | .710 | | .935 |
| 4Q3 | 75.308 | | | | 496.078 | | .852 | | .830 | | .934 |
| 4Q4 | 75.495 | | | | 512.421 | | .783 | | .680 | | .935 |
| 4Q5 | 75.295 | | | | 516.454 | | .731 | | .650 | | .936 |
| 4Q6 | 75.166 | | | | 500.098 | | .845 | | .780 | | .934 |
| 4Q7 | 75.525 | | | | 501.645 | | .804 | | .793 | | .934 |
| 4Q8 | 75.342 | | | | 501.437 | | .860 | | .800 | | .934 |
| 4Q9 | 75.366 | | | | 557.995 | | .111 | | .098 | | .942 |
| 4Q10 | 75.722 | | | | 577.501 | | -.245 | | .311 | | .946 |
| 4Q11 | 75.573 | | | | 501.075 | | .864 | | .822 | | .934 |
| 4Q12 | 75.366 | | | | 522.444 | | .653 | | .482 | | .937 |
| 4Q13 | 75.515 | | | | 547.632 | | .278 | | .304 | | .941 |
| 4Q14 | 75.393 | | | | 533.015 | | .526 | | .513 | | .938 |
| 4Q15 | 75.488 | | | | 522.863 | | .675 | | .550 | | .937 |
| 4Q16 | 75.366 | | | | 498.444 | | .771 | | .731 | | .935 |
| 4Q17 | 75.519 | | | | 500.319 | | .805 | | .765 | | .934 |
| 4Q18 | 75.634 | | | | 541.444 | | .316 | | .288 | | .941 |
| 4Q19 | 75.251 | | | | 526.896 | | .619 | | .510 | | .937 |
| 4Q20 | 75.627 | | | | 565.949 | | -.049 | | .193 | | .944 |
| 4Q21 | 75.353 | | | | 495.494 | | .886 | | .877 | | .933 |
| 4Q22 | 75.434 | | | | 517.131 | | .696 | | .553 | | .936 |
| 4Q23 | 75.278 | | | | 570.508 | | -.133 | | .220 | | .945 |
| 4Q24 | 75.875 | | | | 540.716 | | .414 | | .407 | | .939 |
| 4Q25 | 75.671 | | | | 544.793 | | .307 | | .286 | | .941 |
| 4Q26 | 75.759 | | | | 510.211 | | .811 | | .787 | | .935 |
| **Scale Statistics** | | | | | | | |  |  |  |  |
| Mean | | Variance | | Std. Deviation | | N of Items | |  |  |  |  |
| 78.485 | | 564.598 | | 23.7613 | | 26 | |  |  |  |  |

**Factor Analysis**

| **Total Variance Explained** | | | | | | | | | |
| --- | --- | --- | --- | --- | --- | --- | --- | --- | --- |
| Component | Initial Eigenvalues | | | Extraction Sums of Squared Loadings | | | Rotation Sums of Squared Loadings | | |
|  | Total | % of Variance | Cumulative % | Total | % of Variance | Cumulative % | Total | % of Variance | Cumulative % |
| 1 | 12.451 | 47.889 | 47.889 | 12.451 | 47.889 | 47.889 | 12.354 | 47.515 | 47.515 |
| 2 | 1.902 | 7.316 | 55.205 | 1.902 | 7.316 | 55.205 | 1.671 | 6.427 | 53.942 |
| 3 | 1.290 | 4.962 | 60.167 | 1.290 | 4.962 | 60.167 | 1.380 | 5.306 | 59.248 |
| 4 | 1.148 | 4.415 | 64.581 | 1.148 | 4.415 | 64.581 | 1.338 | 5.145 | 64.393 |
| 5 | 1.066 | 4.100 | 68.682 | 1.066 | 4.100 | 68.682 | 1.115 | 4.289 | 68.682 |
| 6 | .897 | 3.450 | 72.132 |  |  |  |  |  |  |
| 7 | .734 | 2.823 | 74.956 |  |  |  |  |  |  |
| 8 | .687 | 2.642 | 77.598 |  |  |  |  |  |  |
| 9 | .656 | 2.522 | 80.120 |  |  |  |  |  |  |
| 10 | .612 | 2.355 | 82.475 |  |  |  |  |  |  |
| 11 | .520 | 2.001 | 84.476 |  |  |  |  |  |  |
| 12 | .511 | 1.966 | 86.442 |  |  |  |  |  |  |
| 13 | .495 | 1.902 | 88.344 |  |  |  |  |  |  |
| 14 | .410 | 1.578 | 89.922 |  |  |  |  |  |  |
| 15 | .388 | 1.493 | 91.415 |  |  |  |  |  |  |
| 16 | .373 | 1.433 | 92.848 |  |  |  |  |  |  |
| 17 | .321 | 1.235 | 94.083 |  |  |  |  |  |  |
| 18 | .299 | 1.150 | 95.234 |  |  |  |  |  |  |
| 19 | .225 | .867 | 96.101 |  |  |  |  |  |  |
| 20 | .202 | .778 | 96.878 |  |  |  |  |  |  |
| 21 | .178 | .684 | 97.562 |  |  |  |  |  |  |
| 22 | .168 | .647 | 98.209 |  |  |  |  |  |  |
| 23 | .142 | .548 | 98.757 |  |  |  |  |  |  |
| 24 | .129 | .496 | 99.253 |  |  |  |  |  |  |
| 25 | .107 | .410 | 99.663 |  |  |  |  |  |  |
| 26 | .088 | .337 | 100.000 |  |  |  |  |  |  |
| Extraction Method: Principal Component Analysis. | | | | | | | | | |

| **Rotated Component Matrix^a^** | | | | | |
| --- | --- | --- | --- | --- | --- |
|  | Component | | | | |
|  | 1 | 2 | 3 | 4 | 5 |
| 4Q1 | .935 | -.099 | .088 | -.006 | .005 |
| 4Q21 | .927 | -.039 | .084 | .006 | -.036 |
| 4Q11 | .908 | .003 | .003 | -.014 | -.011 |
| 4Q3 | .896 | -.049 | .050 | -.026 | .029 |
| 4Q8 | .871 | .050 | .193 | -.018 | .078 |
| 4Q6 | .860 | .116 | .128 | -.049 | .015 |
| 4Q7 | .844 | .065 | .166 | -.090 | -.046 |
| 4Q17 | .843 | .049 | .050 | -.055 | .006 |
| 4Q16 | .829 | -.012 | -.066 | -.023 | -.026 |
| 4Q2 | .823 | -.002 | -.046 | .047 | .010 |
| 4Q4 | .820 | .078 | -.091 | -.009 | .102 |
| 4Q26 | .816 | .235 | .197 | -.090 | .024 |
| 4Q5 | .776 | -.142 | .060 | .067 | .074 |
| 4Q22 | .725 | -.068 | .133 | .128 | .103 |
| 4Q15 | .719 | .029 | -.281 | .101 | .196 |
| 4Q12 | .677 | .085 | -.010 | .093 | .113 |
| 4Q19 | .667 | -.228 | .091 | .210 | -.017 |
| 4Q14 | .587 | .136 | -.579 | .157 | .096 |
| 4Q24 | .369 | .637 | .123 | .057 | -.029 |
| 4Q23 | -.190 | .592 | -.082 | .113 | -.190 |
| 4Q10 | -.333 | .589 | -.151 | .173 | .137 |
| 4Q25 | .248 | .525 | .349 | -.129 | .288 |
| 4Q13 | .213 | .043 | .741 | .267 | .043 |
| 4Q20 | -.119 | .050 | .005 | .844 | .068 |
| 4Q18 | .255 | .293 | .257 | .597 | -.116 |
| 4Q9 | .068 | -.028 | .005 | .017 | .926 |
| Extraction Method: Principal Component Analysis; Rotation Method: Varimax with Kaiser Normalization;  a. Rotation converged in 6 iterations. | | | | | |
|  | | | | | |

*The exploratory factor analysis indicated that the 26 items that were used to measure Perception categorized into five latent factors for perception and knowledge on antimicrobials. It can be concluded that training has the most influence on the perceptions of and antimicrobial use by future antimicrobial prescribers in animal health compared with any other factor and effort should be intensified to deliver a carefully packaged training targeted at students that takes consideration of the local legislations and other factors into its design.*

**Appendix 1b. Factor extraction using principal component analysis (PCA) method and converged using Varimax Rotation Method with Kaiser Normalization (VRM-KN) for the 10-item questions on students’ depth of knowledge on antimicrobials.**

| **Reliability Statistics** | | | | | |  |  |
| --- | --- | --- | --- | --- | --- | --- | --- |
| Cronbach's Alpha | | Cronbach's Alpha Based on Standardized Items | | N of Items | |  |  |
| .674 | | .705 | | 10 | |  |  |
| **Item-Total Statistics** | | | | | | | |
|  | Scale Mean if Item Deleted | | Scale Variance if Item Deleted | Corrected Item-Total Correlation | Squared Multiple Correlation | | Cronbach's Alpha if Item Deleted |
| 5Q1 | 14.797 | | 22.588 | .456 | .307 | | .635 |
| 5Q2 | 14.605 | | 23.259 | .269 | .153 | | .662 |
| 5Q3 | 14.894 | | 22.663 | .241 | .206 | | .671 |
| 5Q4 | 14.936 | | 23.009 | .336 | .202 | | .651 |
| 5Q5 | 14.614 | | 23.967 | .158 | .151 | | .683 |
| 5Q6 | 14.801 | | 22.676 | .361 | .165 | | .647 |
| 5Q7 | 15.100 | | 22.729 | .556 | .395 | | .628 |
| 5Q8 | 14.785 | | 20.337 | .294 | .173 | | .672 |
| 5Q9 | 14.768 | | 20.669 | .481 | .384 | | .620 |
| 5Q10 | 14.695 | | 21.219 | .414 | .243 | | .634 |

**Factor Analysis**

| **Total Variance Explained** | | | | | | | | | |
| --- | --- | --- | --- | --- | --- | --- | --- | --- | --- |
| Component | Initial Eigenvalues | | | Extraction Sums of Squared Loadings | | | Rotation Sums of Squared Loadings | | |
|  | Total | % of Variance | Cumulative % | Total | % of Variance | Cumulative % | Total | % of Variance | Cumulative % |
| 1 | 2.883 | 28.826 | 28.826 | 2.883 | 28.826 | 28.826 | 1.904 | 19.039 | 19.039 |
| 2 | 1.257 | 12.574 | 41.400 | 1.257 | 12.574 | 41.400 | 1.689 | 16.890 | 35.929 |
| 3 | 1.143 | 11.431 | 52.830 | 1.143 | 11.431 | 52.830 | 1.452 | 14.523 | 50.452 |
| 4 | 1.065 | 10.651 | 63.482 | 1.065 | 10.651 | 63.482 | 1.303 | 13.030 | 63.482 |
| 5 | .742 | 7.422 | 70.903 |  |  |  |  |  |  |
| 6 | .728 | 7.281 | 78.184 |  |  |  |  |  |  |
| 7 | .674 | 6.744 | 84.927 |  |  |  |  |  |  |
| 8 | .627 | 6.274 | 91.202 |  |  |  |  |  |  |
| 9 | .469 | 4.694 | 95.896 |  |  |  |  |  |  |
| 10 | .410 | 4.104 | 100.000 |  |  |  |  |  |  |
| Extraction Method: Principal Component Analysis. | | | | | | | | | |

| **Rotated Component Matrix^a^** | | | | |
| --- | --- | --- | --- | --- |
|  | Component | | | |
|  | 1 | 2 | 3 | 4 |
| 5Q8 | .780 | -.101 | -.064 | .151 |
| 5Q10 | .649 | .268 | .102 | -.049 |
| 5Q7 | .618 | .427 | .178 | .046 |
| 5Q2 | .092 | .763 | -.164 | .042 |
| 5Q1 | .384 | .590 | .191 | -.007 |
| 5Q6 | -.009 | .557 | .365 | .217 |
| 5Q3 | -.025 | -.055 | .876 | .152 |
| 5Q9 | .425 | .291 | .632 | -.162 |
| 5Q5 | -.130 | .279 | -.041 | .811 |
| 5Q4 | .371 | -.134 | .203 | .721 |
| Extraction Method: Principal Component Analysis; Rotation Method: Varimax with Kaiser Normalization. | | | | |
| a. Rotation converged in 10 iterations. | | | | |

*The exploratory factor analysis indicated that the 10 items that were used to measure students’ depth of knowledge on antimicrobials. It can be concluded that almost all the spheres and fields tested influenced the knowledge of and antimicrobial and will determine the use by future antimicrobial prescribers in animal health. Hence, no field of knowledge on antimicrobials can be left out in the training of veterinary students.*
